# Redox Imbalance in Coronary Artery Bypass Grafting: The Clinical Significance of Aminothiols

**DOI:** 10.7759/cureus.96384

**Published:** 2025-11-08

**Authors:** Abubakar I Sidik, Vladislav Dontsov, Maxim L Khavandeev, Dmitriy Sobolev, Malik K Al-Ariki, Debraj Ghosh, Oralee B Pereira, Emmanuel K Kamara, Lyudmila S Korjueva, Mohamed A Cherjaoui, Eissa Mohammed A Ghaleb

**Affiliations:** 1 Cardiovascular Surgery, Peoples' Friendship University of Russia, Moscow, RUS; 2 Cardiothoracic Surgery, Moscow Regional Research and Clinical Institute, Moscow, RUS; 3 Cardiovascular Surgery, Gusak Institute of Emergency and Reconstructive Surgery, Donetsk, RUS; 4 Cardiology, European Medical Center, Moscow, RUS; 5 Pediatric Surgery, Peoples' Friendship University of Russia, Moscow, RUS; 6 Cardiothoracic Surgery, Peoples' Friendship University of Russia, Moscow, RUS; 7 Cardiovascular Medicine, Peoples' Friendship University of Russia, Moscow, RUS

**Keywords:** aminothiols, biomarkers, coronary artery bypass graft, cysteine, cysteinylglycine, glutathione, homocysteine, ischemia-reperfusion injury, oxidative stress, reactive oxygen species

## Abstract

Coronary artery bypass grafting (CABG) is a vital intervention for severe coronary artery disease, yet it induces significant oxidative stress, contributing to postoperative complications and long-term cardiovascular risks. Aminothiols, including glutathione, homocysteine, cysteine, and cysteinylglycine, are key regulators of redox homeostasis, making them promising biomarkers for assessing oxidative stress in CABG patients. This narrative review explores their biochemical roles, measurement techniques, and associations with clinical outcomes. Glutathione mitigates reactive oxygen species (ROS) through the glutathione redox cycle, while elevated homocysteine promotes oxidative damage and inflammation, linked to complications like atrial fibrillation and acute kidney injury. Cysteine and cysteinylglycine further support redox balance, with their dysregulation reflecting perioperative stress. Studies demonstrate that altered aminothiol levels pre- and post-CABG correlate with adverse outcomes, including myocardial injury, graft restenosis, and major adverse cardiovascular events. Advanced analytical methods like high-performance liquid chromatography and liquid chromatography-mass spectrometry enable precise aminothiol quantification, although variability and lack of standardization pose challenges. Therapeutic interventions, such as N-acetylcysteine and folate supplementation, show potential to modulate aminothiol levels and reduce oxidative stress. Despite these insights, gaps in longitudinal data and assay standardization limit clinical adoption. Future research should focus on standardized protocols, large-scale studies, and metabolomics to integrate aminothiols into personalized medicine, enhancing risk stratification and therapeutic strategies for improved CABG outcomes. Overall, this narrative review synthesizes available data on aminothiols and oxidative stress in CABG, with the objective of identifying knowledge gaps and potential pathways for translational and clinical application.

## Introduction and background

Coronary artery bypass grafting (CABG) is a critical surgical procedure for patients with severe coronary artery disease, designed to restore myocardial perfusion by bypassing stenotic arteries with grafts. It remains a standard treatment for patients with multivessel coronary artery disease or left main coronary artery stenosis, improving symptoms and reducing mortality risk [1]. Despite improvements in surgical techniques, CABG induces significant physiological stress, particularly through oxidative mechanisms that impact postoperative recovery [2].

Oxidative stress, characterized by an imbalance between reactive oxygen species (ROS) production and antioxidant defenses, is a key factor in cardiovascular surgery [3]. During CABG, oxidative stress arises from ischemia-reperfusion injury (IRI), cardiopulmonary bypass, and systemic inflammation, leading to cellular damage that can contribute to complications such as postoperative atrial fibrillation and myocardial injury [4]. Identifying biomarkers that reflect oxidative stress is essential for predicting and managing adverse outcomes in CABG patients [5].

Aminothiols, such as glutathione, cysteine, homocysteine, and cysteinylglycine, are sulfur-containing molecules that play a vital role in maintaining redox homeostasis. Glutathione, a primary antioxidant, neutralizes ROS and protects cells from oxidative damage, while homocysteine and cysteine are involved in redox signaling and metabolic pathways [6]. Dysregulated aminothiol levels, particularly elevated homocysteine or depleted glutathione, have been associated with cardiovascular pathology and increased oxidative stress [7]. In CABG, these molecules are promising biomarkers due to their responsiveness to perioperative oxidative changes.

The rationale for studying aminothiols in CABG lies in their potential to serve as sensitive indicators of oxidative stress and predictors of clinical outcomes. For instance, altered glutathione levels may reflect oxidative burden, while elevated homocysteine has been linked to endothelial dysfunction and adverse cardiovascular events [8]. Measuring aminothiol profiles could enhance risk stratification and guide interventions to mitigate oxidative stress, such as antioxidant therapies, to improve patient outcomes [9]. This narrative review aims to synthesize evidence on the role of aminothiols as biomarkers of oxidative stress and their association with clinical outcomes in CABG patients. By evaluating the biochemical and clinical significance of aminothiols, we seek to highlight their potential in perioperative care and identify areas for future research.

## Review

Methods

This narrative review employs a non-systematic, expert-driven synthesis of the literature to explore the role of aminothiols as biomarkers of oxidative stress and their association with clinical outcomes in patients undergoing CABG. The narrative approach allows for a comprehensive and interpretive overview of the topic, integrating insights from diverse studies to provide a cohesive understanding [10]. This method is particularly suited for synthesizing complex and heterogeneous data on aminothiols and oxidative stress in the context of CABG.

Literature was sourced from peer-reviewed scientific databases, including PubMed, Scopus, and Web of Science, to ensure access to high-quality, relevant publications. Additional searches were conducted using Google Scholar to identify grey literature, such as conference abstracts or theses, that might provide supplementary insights. The search strategy utilized keywords and MeSH terms such as “aminothiols,” “glutathione,” “homocysteine,” “cysteine,” “oxidative stress,” “coronary artery bypass grafting,” “CABG,” “biomarkers,” and “clinical outcomes,” combined with Boolean operators (AND, OR) to refine results.

Studies were selected based on specific inclusion criteria: (1) focus on aminothiols (e.g., glutathione, homocysteine, cysteine, or cysteinylglycine) as biomarkers of oxidative stress, (2) relevance to CABG procedures, and (3) reporting of clinical outcomes, such as postoperative complications, graft patency, or long-term cardiovascular events. Studies were excluded if they focused exclusively on non-CABG cardiac surgeries, lacked data on aminothiols, or were not published in English. Both human and relevant animal studies were considered, with priority given to clinical studies involving CABG patients.

The timeframe for included studies spans from January 2000 to October 2025, capturing the past 25 years to reflect advancements in CABG techniques, biomarker assays, and oxidative stress research [11]. This timeframe ensures the inclusion of contemporary data while accounting for foundational studies that established the role of aminothiols in redox biology.

The evidence was synthesized qualitatively, without meta-analysis, due to the heterogeneity of study designs, aminothiol measurement techniques, and reported outcomes [12]. This approach involved critical appraisal of the literature to identify patterns, gaps, and inconsistencies, with an emphasis on integrating biochemical and clinical findings to elucidate the role of aminothiols in CABG. Key studies were evaluated for methodological rigor, including sample size, study design, and assay reliability, to ensure the validity of the conclusions drawn.

Pathophysiology of oxidative stress

Mechanisms of ROS Generation During CABG

During CABG, ROS generation is primarily driven by IRI and cardiopulmonary bypass (CPB). Ischemia-reperfusion injury occurs when blood flow is restored to ischemic myocardial tissue, leading to a burst of ROS production from mitochondria, xanthine oxidase, and nicotinamide adenine dinucleotide phosphate (reduced form) (NADPH) oxidase [13]. This process is exacerbated during CABG due to aortic cross-clamping and cardioplegic arrest, which induce transient ischemia followed by reperfusion upon graft placement [14]. CPB further amplifies ROS production by activating neutrophils, which release superoxide and hydrogen peroxide, and by exposing blood to non-physiological surfaces, triggering inflammatory cascades [4]. These mechanisms collectively increase oxidative stress, overwhelming endogenous antioxidant systems and causing cellular damage.

**Figure 1:**
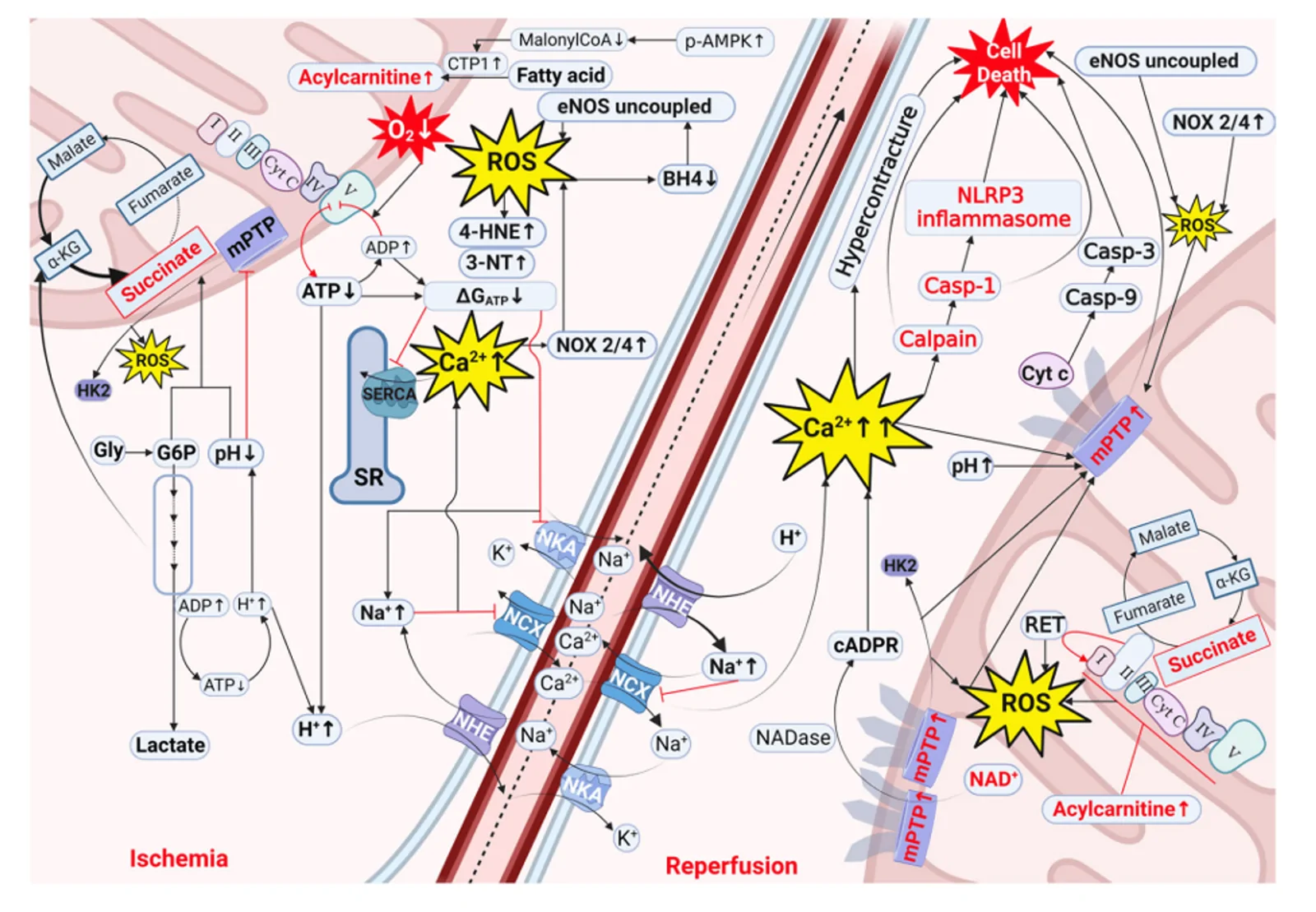
Schematic diagram of mechanisms during the ischemic and reperfusion period that together cause acute cardiac ischemia-reperfusion injury (IRI). The figure illustrates key processes including decrease in Gibbs free energy of adenosine triphosphate (ΔGATP), increased acidosis, sodium ion (Na+) and calcium ion (Ca2+) build-up, succinate accumulation, hexokinase II (HKII) detachment, acylcarnitine accumulation, tetrahydrobiopterin (BH4) oxidation, and reactive oxygen species (ROS) production via reverse electron transport (RET), NADPH oxidase (NOX), and endothelial nitric oxide synthase (eNOS) uncoupling, leading to ischemia-reperfusion injury (IRI) in coronary artery bypass grafting (CABG). Reprinted under the terms of the Creative Commons Attribution 4.0 International License from Wang et al. [15]. NADPH: nicotinamide adenine dinucleotide phosphate (reduced form); α-KG: α-ketoglutarate; mPTP: mitochondrial permeability transition pore; HK2: hexokinase 2; ATP: adenosine triphosphate; ADP: adenosine diphosphate; CPT1: carnitine palmitoyltransferase 1; BH4: tetrahydrobiopterin; p-AMPK: phosphorylated AMP-activated protein kinase; 4-HNE: 4-hydroxynonenal; 3-NT: 3-nitrotyrosine; SR: sarcoplasmic reticulum; NLRP3: NOD-, LRR-, and pyrin domain-containing protein 3 (inflammasome); Casp: Caspase; cADPR: cyclic ADP-ribose; NAD: nicotinamide adenine dinucleotide; NKA: Na⁺/K⁺-ATPase (sodium-potassium pump); NCX: sodium-calcium exchanger; NHE: sodium-hydrogen exchanger.

The mechanisms (IRI and CPB) that lead to the generation of ROS during CABG involve mitochondrial succinate accumulation, hexokinase II (HKII) detachment, NADPH oxidase (NOX) activation, and endothelial nitric oxide synthase (eNOS) uncoupling, which collectively overwhelm antioxidant defenses and contribute to cellular damage. To illustrate these processes, Figure 1 provides a schematic diagram of the mechanisms during the ischemic and reperfusion periods that cause acute cardiac IRI, highlighting the interplay of metabolic changes, ion imbalances, and ROS production that underpin oxidative stress in CABG [15].

**Figure 2:**
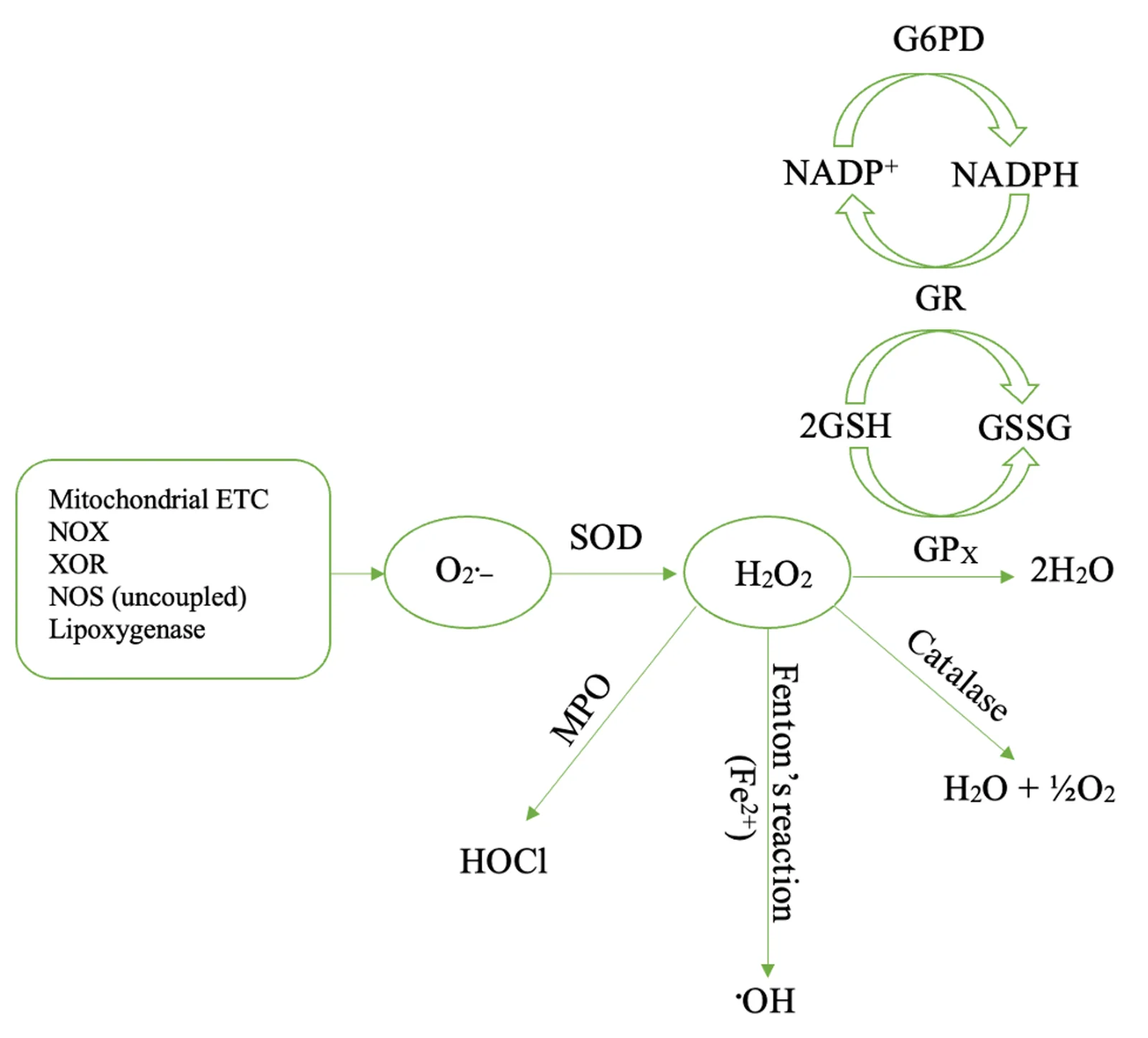
Diagram of reactive oxygen species (ROS) sources and detoxification pathways. The figure illustrates ROS sources such as NADPH oxidase, xanthine oxidase, mitochondrial electron transport chain (ETC), and myeloperoxidase (MPO) from neutrophils, along with detoxification mechanisms including superoxide dismutase (SOD), glutathione peroxidase (GPx), catalase, and glutathione (GSH) cycle, showing the imbalance leading to hydrogen peroxide (H₂O₂)/hypochlorous acid (HOCl)/hydroxyl radical (•OH) causing lipid/protein/deoxyribonucleic acid (DNA) damage in ischemia-reperfusion (I/R) contexts relevant to coronary artery bypass grafting (CABG). Image credit: Authors. NADPH: nicotinamide adenine dinucleotide phosphate (reduced form); G6PD: glucose-6-phosphate dehydrogenase; NADP: nicotinamide adenine dinucleotide phosphate (oxidized form); GR: glutathione reductase; GSSG: oxidized glutathione; NOX: NADPH oxidase; XOR: xanthine oxidoreductase; NOS: nitric oxide synthase.

Oxidative challenges, if not countered by robust antioxidant mechanisms, can significantly influence the severity of injury in cardiovascular conditions, highlighting the need for targeted therapeutic strategies to restore redox homeostasis [16]. Figure 2 provides a diagram of ROS sources and detoxification pathways, highlighting the imbalance that exacerbates oxidative stress in CABG.

Impact of Oxidative Stress on Myocardial Tissue, Endothelial Function, and Systemic Inflammation

**Figure 3:**
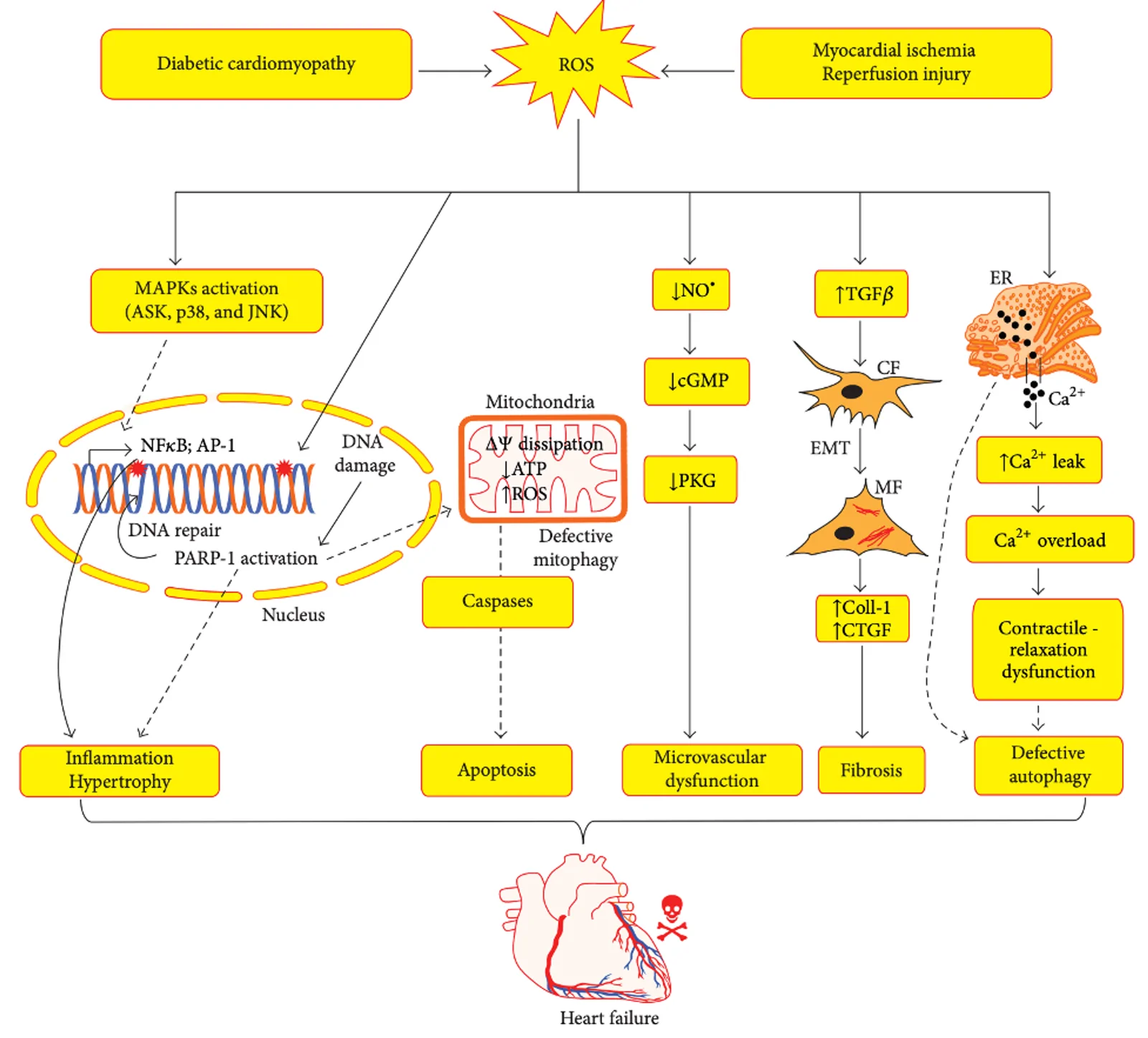
Schematic diagram of reactive oxygen species (ROS) in myocardial ischemia-reperfusion (I/R). The figure illustrates ROS-mediated impacts, including endothelial nitric oxide (NO) depletion leading to peroxynitrite (ONOO⁻) formation, inflammation via nuclear factor-kappa B (NF-κB) and cytokines (e.g., tumor necrosis factor-alpha (TNF-α), interleukin-6 (IL-6)), myocardial injury through apoptosis and stunning, and fibrosis via remodeling, contributing to oxidative stress effects on myocardial tissue, endothelial function, and systemic inflammation in coronary artery bypass grafting (CABG). Reprinted under the terms of the Creative Commons Attribution 4.0 International License from Kuria et al. [16]. MAPK: mitogen-activated protein kinase; ASK: apoptosis signal-regulating kinase; JNK: c-Jun N-terminal kinase; cGMP: cyclic guanosine monophosphate; PKG: protein kinase G; CF: cardiac fibroblast; EMT: epithelial-to-mesenchymal transition; MF: myofibroblast; CTGF: connective tissue growth factor; PARP-1: poly(ADP-ribose) polymerase-1; AP-1: activator protein-1.

Oxidative stress during CABG has profound effects on myocardial tissue, endothelial function, and systemic inflammation. In myocardial tissue, ROS cause lipid peroxidation, protein oxidation, and DNA damage, leading to myocyte apoptosis and impaired contractility [17]. This oxidative damage is particularly pronounced during reperfusion, contributing to myocardial stunning and reduced cardiac output [13]. Endothelial function is compromised as ROS impair nitric oxide bioavailability, promoting vasoconstriction and endothelial dysfunction, which can hinder graft patency and increase thrombotic risk [18]. Systemically, oxidative stress triggers an inflammatory response by activating pro-inflammatory cytokines (e.g., tumor necrosis factor (TNF) α, interleukin (IL) 6) and upregulating adhesion molecules, exacerbating tissue injury and multi-organ dysfunction [19]. These effects underscore the widespread impact of oxidative stress in CABG patients. The interplay of oxidative and reductive stress in myocardial ischemia-reperfusion (I/R) injury significantly influences cardiac remodeling, where ROS contribute to endothelial dysfunction and inflammation, subsequently promoting fibrosis and myocyte apoptosis. Kurian et al. note that this oxidative burden not only exacerbates acute injury but also drives long-term pathological remodeling, suggesting that therapeutic interventions targeting ROS and the restoration of proteostasis could mitigate the progression of heart failure in conditions like CABG [16]. To illustrate these impacts, Figure 3 provides a schematic of ROS in myocardial I/R, highlighting the pathways to endothelial dysfunction, inflammation, myocardial injury, and fibrosis in CABG.

Clinical Implications

Association of oxidative stress with postoperative complications: Oxidative stress is strongly associated with postoperative complications in CABG, including atrial fibrillation, acute kidney injury (AKI), and prolonged ventilation. Postoperative atrial fibrillation, a common complication, is linked to ROS-mediated electrical remodeling and inflammation in atrial tissue, with studies showing elevated oxidative stress markers in affected patients [20]. AKI is another significant concern, as oxidative damage to renal tubular cells, driven by CPB-induced ROS, increases the risk of renal dysfunction, particularly in patients with prolonged bypass times [21]. Prolonged ventilation, often resulting from oxidative stress-induced lung injury, is associated with increased ROS production and reduced antioxidant capacity in the pulmonary vasculature [22]. These complications highlight the role of oxidative stress in driving adverse short-term outcomes.

Long-term effects on patient recovery and cardiovascular events: The long-term effects of oxidative stress in CABG patients include impaired recovery and increased risk of cardiovascular events. Persistent oxidative stress contributes to endothelial dysfunction and neointimal hyperplasia, which can lead to graft restenosis and reduced long-term graft patency [23]. Oxidative stress also promotes atherosclerosis progression, increasing the incidence of major adverse cardiovascular events (MACE), such as myocardial infarction and stroke, in the years following CABG [24]. Furthermore, systemic inflammation driven by oxidative stress can exacerbate comorbidities like diabetes and hypertension, negatively impacting overall recovery and survival [8]. These long-term effects emphasize the need for strategies to mitigate oxidative stress to improve patient outcomes.

Biochemical role of aminothiols in oxidative stress

**Figure 4:**
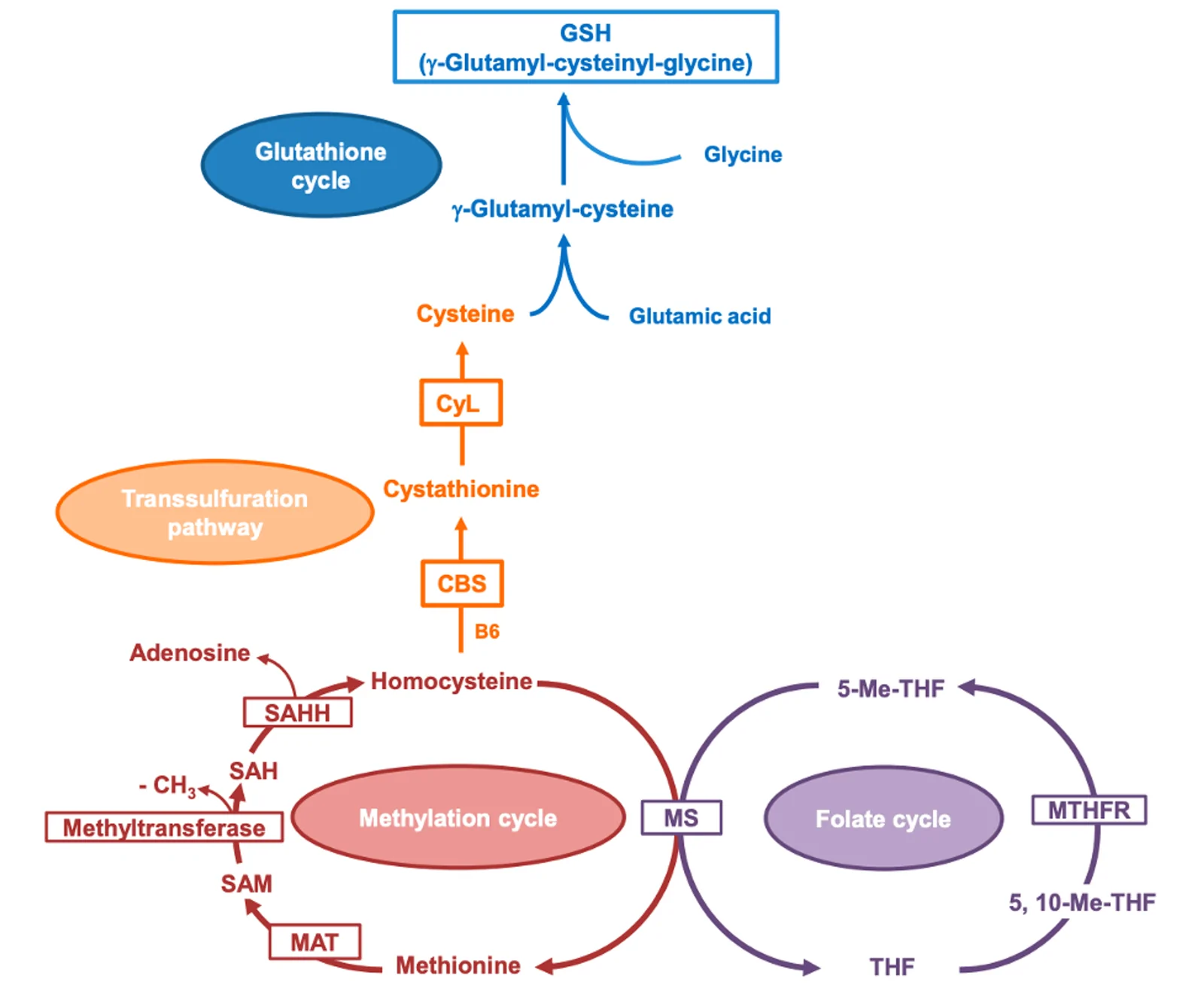
Flowchart of homocysteine metabolism intersecting glutathione (GSH) production/folate cycle. The figure illustrates the biochemical pathways of aminothiols, showing homocysteine (Hcy) metabolism via the methionine cycle and transsulfuration pathway (cystathionine β-synthase (CBS)/cystathionine γ-lyase (CyL)) to cysteine (Cys), which serves as a precursor for glutathione (GSH) synthesis, along with glutathione (GSH) degradation to cysteinylglycine (CysGly) via gamma-glutamyl transferase (GGT), and the folate (THF) cycle involving 5-methyltetrahydrofolate (5-MeTHF) and 5,10-methylenetetrahydrofolate (5,10-MeTHF) by methylenetetrahydrofolate reductase (MTHFR), highlighting their roles in maintaining redox homeostasis in coronary artery bypass grafting (CABG). Reprinted under the terms of the Creative Commons Attribution 4.0 International License from Pastore et al. [28]. SAHH: S-adenosylhomocysteine hydrolase; SAH: S-adenosylhomocysteine; MAT: methionine adenosyltransferase; MS: methionine synthase.

Aminothiols are a group of sulfur-containing compounds critical to cellular redox homeostasis, including cysteine, glutathione, homocysteine, and cysteinylglycine. Cysteine serves as a precursor for glutathione synthesis and participates in protein structure stabilization through disulfide bonds [25]. Glutathione, a tripeptide composed of cysteine, glutamate, and glycine, exists in reduced (GSH) and oxidized (GSSG) forms, functioning as a primary intracellular antioxidant [26]. Homocysteine, a byproduct of methionine metabolism, is implicated in redox signaling and vascular pathology when elevated [7]. Cysteinylglycine, a glutathione degradation product, also contributes to antioxidant defense [27]. These molecules collectively maintain cellular redox balance by modulating oxidative and reductive processes. Pastore et al. highlight that this metabolic network is central to maintaining redox homeostasis, with disruptions leading to oxidative stress and related pathologies [28]. Figure 4 provides a flowchart depicting homocysteine metabolism intersecting with glutathione production and the folate cycle, emphasizing enzymatic reactions like cystathionine β-synthase (CBS) and cystathionine γ-lyase (CyL) in the transsulfuration pathway, and their role in neutralizing ROS in the context of CABG.

Aminothiols play a pivotal role in combating oxidative stress, defined as an imbalance favoring ROS production over antioxidant defenses. Glutathione, in its reduced form (GSH), directly scavenges ROS, such as superoxide and hydrogen peroxide, thereby protecting cellular components from oxidative damage [26]. The GSH/GSSG ratio is a key indicator of redox status, with a higher ratio reflecting greater antioxidant capacity [6]. Cysteine supports glutathione synthesis and can directly reduce ROS, while homocysteine, at physiological levels, contributes to redox signaling but becomes pro-oxidative when elevated [7]. Cysteinylglycine, though less studied, aids in extracellular redox buffering [27]. These actions collectively mitigate oxidative stress, which is critical in high-stress conditions like surgery.

The mechanisms by which aminothiols interact with ROS involve both enzymatic and non-enzymatic pathways. Glutathione neutralizes ROS via direct reduction, facilitated by enzymes such as glutathione peroxidase, which converts hydrogen peroxide to water while oxidizing GSH to GSSG [29]. GSSG is then reduced back to GSH by glutathione reductase, utilizing NADPH as a cofactor, thus maintaining the redox cycle [26]. Cysteine can directly scavenge ROS or serve as a substrate for glutathione synthesis, while homocysteine may indirectly influence ROS levels by modulating endothelial nitric oxide synthase activity [30]. These interactions are tightly regulated to prevent oxidative damage to lipids, proteins, and DNA, which can impair cellular function. In the context of cardiovascular disease and surgical stress, aminothiol metabolism is highly relevant. Oxidative stress is a hallmark of cardiovascular diseases, including atherosclerosis, where elevated homocysteine levels promote endothelial dysfunction and ROS production [7]. In CABG, surgical procedures such as cardiopulmonary bypass and IRI exacerbate ROS generation, depleting GSH and altering the GSH/GSSG ratio [4]. These changes can contribute to postoperative complications, such as myocardial injury and atrial fibrillation [20]. Dysregulated aminothiol metabolism, particularly reduced GSH or elevated homocysteine, reflects the oxidative burden and may serve as a prognostic indicator in CABG patients, highlighting the need to explore these molecules as biomarkers [8].

The biochemical roles of aminothiols, including glutathione, homocysteine, cysteine, and cysteinylglycine, are critical to understanding their potential as biomarkers in CABG. These sulfur-containing molecules, each with unique contributions to redox homeostasis and ROS interactions, play pivotal roles in modulating oxidative stress, with implications for perioperative complications and long-term outcomes. To provide a clear overview of these roles and their relevance to CABG, Table 1 summarizes the biochemical functions, impact on oxidative stress, and clinical significance of each aminothiol. This synthesis highlights their interconnected contributions to cellular protection, setting the stage for a detailed exploration of their biomarker applications.

**Table 1:** Overview of Aminothiols and Their Roles in CABG. ROS: reactive oxygen species; GSH: reduced glutathione; GSSG: oxidized glutathione; CPB: cardiopulmonary bypass; MACE: major adverse cardiac events; CABG: coronary artery bypass grafting.

Aminothiol	Biochemical Role	Impact on Oxidative Stress	Clinical Relevance in CABG
Glutathione	Primary antioxidant, scavenges ROS via GSH/GSSG cycle, neutralizes H₂O₂ via glutathione peroxidase [26,28].	Reduces oxidative damage during CPB by detoxifying ROS; maintains redox homeostasis [26,31].	Low GSH/GSSG ratio predicts postoperative myocardial infarction and acute kidney injury [32].
Homocysteine	Byproduct of methionine metabolism, modulates redox signaling at physiological levels [28,33].	Elevated levels promote ROS production via auto-oxidation, inhibit antioxidant enzymes [33,34].	High preoperative levels linked to postoperative atrial fibrillation, endothelial dysfunction, and MACE [35].
Cysteine	Precursor for glutathione synthesis, stabilizes protein structure via disulfide bonds [28,29].	Directly scavenges ROS, supports GSH production to counter oxidative stress [31,36].	Depletion post-CABG associated with increased oxidative burden and myocardial injury [36].
Cysteinylglycine	Glutathione degradation product, contributes to extracellular redox buffering [26,28].	Aids in neutralizing extracellular ROS, supports redox balance during surgical stress [31].	Less studied; potential marker for oxidative stress and postoperative complications [37].

Aminothiols as biomarkers in CABG

Aminothiols, including plasma glutathione (GSH and GSSG), homocysteine, cysteine, and cysteinylglycine, have been investigated as potential biomarkers of oxidative stress in patients undergoing CABG. Plasma glutathione, particularly the reduced form (GSH) and its oxidized counterpart (GSSG), is a critical indicator of cellular redox status due to its role as a primary antioxidant [26]. Homocysteine, a sulfur-containing amino acid, has been extensively studied for its association with cardiovascular risk and oxidative stress [7]. Cysteine, a precursor to glutathione, and cysteinylglycine, a glutathione degradation product, are less frequently examined but contribute to the redox environment in CABG patients [27]. These aminothiols are of interest due to their sensitivity to the oxidative stress induced by CABG procedures.

Measurement of aminothiol levels in clinical settings typically involves advanced analytical techniques to ensure accuracy and sensitivity. High-performance liquid chromatography (HPLC) coupled with fluorescence detection or ultraviolet absorbance is commonly used to quantify plasma GSH, GSSG, homocysteine, and cysteine levels due to its high resolution and ability to separate thiol compounds [38]. Mass spectrometry (MS), often combined with liquid chromatography (LC-MS), offers greater specificity and sensitivity, particularly for detecting low-abundance thiols like cysteinylglycine, and is increasingly utilized in research settings [39]. Enzyme-linked immunosorbent assays (ELISA) are also employed for homocysteine measurement in clinical practice due to their accessibility, although they may lack the precision of HPLC or MS for other aminothiols [40]. These methods require careful sample preparation to prevent thiol oxidation, which can skew results [38]. Table 2 summarizes these measurement techniques, detailing the aminothiols measured, their advantages, limitations, and clinical applicability, providing a concise reference for their role in research and potential integration into clinical practice.

**Table 2:** Measurement Techniques for Aminothiols in CABG. HPLC: high-performance liquid chromatography; LC-MS: liquid chromatography-mass spectrometry; ELISA: enzyme-linked immunosorbent assay; GSH: reduced glutathione; GSSG: oxidized glutathione.

Technique	Aminothiol Measured	Advantages	Limitations	Clinical Applicability
HPLC [37]	GSH, GSSG, homocysteine, cysteine	High resolution, separates thiols effectively.	Requires careful sample handling to prevent oxidation, time-consuming derivatization.	Widely used in research but complex for routine clinical use.
LC-MS [41]	GSH, GSSG, homocysteine, cysteine, cysteinylglycine	High sensitivity and specificity, simultaneous multi-analyte detection.	High cost, requires specialized equipment and expertise.	Emerging in clinical research, limited by cost for routine diagnostics.
ELISA [31]	GSH, homocysteine	Simple, cost-effective, high-throughput for specific analytes.	Lower precision for complex mixtures, prone to cross-reactivity.	Suitable for rapid screening but less reliable for precise quantification.

Nolin et al. demonstrate a robust HPLC method that reduces plasma aminothiols with tris-(2-carboxyethyl)-phosphine hydrochloride, precipitates proteins with trichloroacetic acid, and derivatizes with ammonium-7-fluorobenzo-2-oxa-1,3-diazole-4-sulfonic acid for fluorescence detection [37]. Evidence linking aminothiol levels to oxidative stress during CABG is robust. Studies have shown that cardiopulmonary bypass and ischemia-reperfusion injury during CABG significantly increase ROS production, leading to a depletion of plasma GSH and an increase in GSSG, reflecting a shift toward oxidative stress [4]. Elevated homocysteine levels pre-CABG have been associated with increased oxidative damage, as homocysteine promotes ROS generation through auto-oxidation and inhibition of antioxidant enzymes [7]. Cysteine levels, while less studied, have been shown to decrease post-CABG, potentially due to its utilization in glutathione synthesis to counter oxidative stress [42]. These changes in aminothiol profiles indicate a direct relationship with the oxidative burden experienced during CABG, making them valuable biomarkers for monitoring perioperative redox status.

Variability in aminothiol levels pre- and post-CABG has significant clinical implications. Preoperatively, reduced GSH levels or elevated homocysteine are associated with higher cardiovascular risk and may predict adverse outcomes, such as postoperative myocardial infarction or atrial fibrillation [8]. Post-CABG, a significant decline in GSH and an increase in GSSG have been observed, correlating with the intensity of oxidative stress and the likelihood of complications like acute kidney injury [4]. Homocysteine levels often remain elevated post-surgery, particularly in patients with prolonged cardiopulmonary bypass, and are linked to endothelial dysfunction and poor long-term outcomes [24]. Variability in these levels may be influenced by patient factors such as age, comorbidities (e.g., diabetes), and preoperative antioxidant status, as well as surgical factors like bypass duration [42]. Monitoring aminothiol dynamics could thus aid in risk stratification and guide interventions, such as antioxidant supplementation, to mitigate oxidative stress and improve clinical outcomes in CABG patients.

Clinical outcomes associated with aminothiols in CABG

Aminothiols, such as glutathione (GSH and GSSG), homocysteine, cysteine, and cysteinylglycine, are promising biomarkers for predicting clinical outcomes in CABG due to their sensitivity to oxidative stress. Their dysregulation is linked to both short-term and long-term outcomes, offering potential for risk stratification and targeted clinical interventions.

Short-Term Outcomes

Postoperative complications: Oxidative stress during CABG contributes to postoperative complications, including myocardial infarction and atrial fibrillation. Elevated preoperative homocysteine levels are associated with an increased risk of postoperative atrial fibrillation, driven by homocysteine’s role in promoting oxidative stress and inflammation, which disrupts atrial electrophysiology [43]. A reduced GSH/GSSG ratio, indicative of oxidative imbalance, is linked to postoperative myocardial infarction, as GSH depletion impairs the neutralization of ROS during ischemia-reperfusion, leading to myocyte damage [32]. These complications increase morbidity and prolong recovery, underscoring the role of aminothiol dysregulation in acute postoperative events.

Acute kidney injury and other organ dysfunction: Acute kidney injury (AKI) and other organ dysfunctions are significant postoperative concerns in CABG, exacerbated by oxidative stress. Low GSH levels during CPB are associated with AKI, as ROS-mediated damage to renal tubular cells impairs kidney function, particularly in patients with extended CPB duration [44]. Oxidative stress also contributes to pulmonary injury, increasing the risk of prolonged ventilation, and can lead to liver dysfunction through systemic redox imbalances [4]. Elevated homocysteine and reduced GSH serve as markers of oxidative burden, reflecting the susceptibility of multiple organs to perioperative stress.

Long-Term Outcomes

Graft patency and restenosis: Long-term graft patency is influenced by oxidative stress and aminothiol dysregulation. Reduced GSH levels promote endothelial dysfunction and neointimal hyperplasia, increasing the risk of graft restenosis by enhancing vascular smooth muscle cell proliferation [45]. Elevated homocysteine levels further exacerbate graft failure by inducing oxidative stress and inflammation, which impair endothelial integrity and promote vascular remodeling [46]. Maintaining balanced aminothiol levels could support long-term graft function and reduce restenosis risk.

Major adverse cardiovascular events (MACE): Adverse events, such as myocardial infarction, stroke, and heart failure, are more frequent in CABG patients with aminothiol dysregulation. Elevated homocysteine levels post-CABG are associated with a higher incidence of MACE, driven by accelerated atherosclerosis and endothelial dysfunction through ROS generation and inflammatory pathways [35]. GSH depletion amplifies this risk by compromising antioxidant defenses, allowing oxidative damage to contribute to cardiovascular events [32].

Mortality rates: Mortality rates following CABG are influenced by persistent aminothiol imbalances. Elevated homocysteine is linked to increased long-term mortality due to its role in promoting atherosclerosis and thrombotic events [35]. Similarly, low GSH levels correlate with higher mortality risk, as impaired antioxidant capacity exacerbates oxidative stress, negatively impacting survival [47]. These associations highlight aminothiols as critical predictors of long-term prognosis in CABG patients.

Studies Correlating Aminothiol Levels With Specific Outcomes

Several studies have established correlations between aminothiol levels and CABG outcomes. Mariscalco et al. (2006) found that preoperative hyperhomocysteinemia was associated with postoperative atrial fibrillation, linking homocysteine to oxidative stress-related complications [43]. Karu et al. (2005) demonstrated that a reduced GSH/GSSG ratio during CABG was associated with myocardial injury and AKI, emphasizing the role of glutathione depletion [32]. Schnyder et al. (2001) reported that elevated homocysteine levels post-CABG predicted MACE and increased mortality, supporting its prognostic value [35]. Pignatelli et al. (2010) showed that cysteine depletion post-CABG correlated with oxidative stress markers, contributing to both short- and long-term complications [47]. These studies underscore the clinical significance of aminothiols in CABG outcomes.

Potential Prognostic Value of Aminothiols in Risk Stratification

Aminothiols offer substantial potential for risk stratification in CABG patients. Preoperative measurement of homocysteine and GSH levels can identify patients at risk for postoperative complications, enabling targeted interventions such as folate supplementation to lower homocysteine or antioxidant therapies to bolster GSH [48]. Perioperative monitoring of the GSH/GSSG ratio could guide strategies to mitigate AKI and myocardial injury, while long-term tracking of aminothiol levels may predict MACE and mortality, informing follow-up care [35]. Integrating aminothiol profiling into predictive models, alongside biomarkers like troponin, could enhance personalized medicine approaches, although standardized assays and large-scale validation studies are needed [49].

The dysregulation of aminothiols, such as elevated homocysteine and reduced GSH/GSSG ratios, is intricately linked to adverse clinical outcomes in CABG, including postoperative complications and long-term cardiovascular events. These associations highlight the potential of aminothiols as prognostic biomarkers for risk stratification and therapeutic targeting. Table 3 summarizes the key clinical outcomes associated with aminothiol dysregulation in CABG, detailing the specific aminothiol changes, supporting evidence from pivotal studies, and underlying mechanisms driving these effects. This overview underscores the critical role of aminothiols in perioperative and long-term management of CABG patients.

**Table 3:** Clinical Outcomes Associated With Aminothiol Dysregulation in CABG. ROS: reactive oxygen species; CPB: cardiopulmonary bypass; GSH: reduced glutathione; GSSG: oxidized glutathione.

Outcome	Associated Aminothiol Change	Evidence from Studies	Potential Mechanisms
Postoperative atrial fibrillation (POAF)	Elevated homocysteine	Patel et al. (2016) linked hyperhomocysteinemia to increased POAF risk in cardiac surgery patients [50].	Promotes oxidative stress and atrial inflammation via ROS production and cytokine activation.
Acute kidney injury (AKI)	Reduced GSH/GSSG ratio	Karu et al. (2005) showed low GSH/GSSG ratio associated with AKI during CABG [32].	ROS-mediated damage to renal tubular cells, exacerbated by CPB-induced oxidative stress.
Graft restenosis	Elevated homocysteine, reduced GSH	Schnyder et al. (2001) reported high homocysteine predicts restenosis; GSH depletion linked to neointimal hyperplasia [35].	Homocysteine induces endothelial dysfunction; GSH depletion promotes vascular smooth muscle proliferation.
Major adverse cardiovascular events (MACE)	Elevated homocysteine, reduced GSH/GSSG ratio	Schnyder et al. (2001) linked homocysteine to MACE; Karu et al. (2005) tied GSH depletion to cardiovascular events [32,35].	Accelerated atherosclerosis via ROS and inflammation; GSH depletion impairs antioxidant defense.
Mortality	Elevated homocysteine, low GSH	Schnyder et al. (2001) and Pignatelli et al. (2010) associated high homocysteine and low GSH with increased mortality [35,36].	Homocysteine promotes thrombosis and atherosclerosis; low GSH exacerbates oxidative damage.

Mechanistic insights

Aminothiols, including glutathione, homocysteine, cysteine, and cysteinylglycine, play a critical role in modulating oxidative stress and interact with various pathways that influence clinical outcomes in CABG. Understanding their mechanistic contributions provides insights into their potential as biomarkers and therapeutic targets.

Aminothiols and Redox Pathways

How aminothiols modulate oxidative stress: Aminothiols are integral to cellular redox homeostasis, with GSH being the primary intracellular antioxidant. GSH neutralizes ROS, such as superoxide and hydrogen peroxide, through direct scavenging and enzymatic reactions catalyzed by glutathione peroxidase, which converts GSH to its oxidized form (GSSG) while reducing ROS [26]. GSSG is subsequently recycled back to GSH by glutathione reductase, maintaining the GSH/GSSG ratio, a key indicator of redox status [6]. Cysteine, a precursor for GSH synthesis, also directly scavenges ROS, while homocysteine and cysteinylglycine contribute to redox balance in extracellular compartments [3]. During CABG, the surge in ROS from ischemia-reperfusion and CPB depletes GSH, shifting the redox balance toward oxidative stress, which aminothiols counteract to protect cellular integrity [27].

Interaction with other biomarkers: Aminothiols interact with other biomarkers, such as inflammatory cytokines and nitric oxide (NO), to modulate oxidative stress and inflammation. Elevated homocysteine levels promote the production of pro-inflammatory cytokines, such as TNF-α and IL-6, which exacerbate oxidative stress by activating NADPH oxidase and increasing ROS production [5]. Conversely, GSH mitigates inflammation by inhibiting cytokine release and stabilizing NO bioavailability, which is critical for endothelial function [9]. Homocysteine can reduce NO levels by generating superoxide, leading to the formation of peroxynitrite, a potent oxidant that further amplifies oxidative damage [4]. These interactions highlight the interconnected roles of aminothiols, cytokines, and NO in the oxidative and inflammatory milieu of CABG.

Pathophysiological Implications

Potential mechanisms linking aminothiol dysregulation to adverse outcomes in CABG: Aminothiol dysregulation, particularly reduced GSH and elevated homocysteine, is mechanistically linked to adverse outcomes in CABG. GSH depletion during CPB and ischemia-reperfusion impairs the neutralization of ROS, leading to oxidative damage to lipids, proteins, and DNA, which contributes to myocardial injury and postoperative complications [51]. Elevated homocysteine promotes oxidative stress by inhibiting antioxidant enzymes like superoxide dismutase and by auto-oxidation, generating ROS that exacerbate cellular damage [8]. These mechanisms increase the risk of adverse outcomes, such as postoperative atrial fibrillation and acute kidney injury, by amplifying oxidative and inflammatory stress [20]. Additionally, cysteinylglycine dysregulation may impair extracellular redox buffering, further contributing to tissue injury [27].

Role of aminothiols in endothelial dysfunction, thrombosis, and myocardial injury: Aminothiols play a significant role in endothelial dysfunction, thrombosis, and myocardial injury, key contributors to CABG complications. Elevated homocysteine induces endothelial dysfunction by reducing NO bioavailability and upregulating adhesion molecules, promoting leukocyte adhesion and vascular inflammation [7]. This dysfunction increases thrombotic risk by enhancing platelet activation and coagulation, potentially leading to graft occlusion [9]. GSH depletion exacerbates myocardial injury by failing to counteract ROS-induced lipid peroxidation and myocyte apoptosis, contributing to myocardial stunning and reduced contractility post-CABG [4]. Cysteine, as a GSH precursor, supports antioxidant defenses, but its depletion during surgery can worsen myocardial damage [42]. These pathophysiological roles underscore the potential of aminothiols as biomarkers for identifying patients at risk of adverse outcomes and as targets for therapeutic intervention.

Clinical applications

The integration of aminothiols, such as glutathione (GSH and GSSG), homocysteine, cysteine, and cysteinylglycine, into clinical practice for CABG patients holds promise for improving diagnostic accuracy, guiding therapeutic interventions, and enabling personalized medicine. Their role as biomarkers of oxidative stress offers opportunities to enhance patient outcomes through targeted strategies.

Diagnostic Potential

Use of aminothiols as preoperative risk stratification tools: Aminothiols can serve as preoperative risk stratification tools by identifying patients at higher risk for adverse outcomes based on their redox status. Elevated preoperative homocysteine levels are associated with increased risks of postoperative complications, such as atrial fibrillation and myocardial infarction, allowing clinicians to identify high-risk individuals for closer monitoring or preventive interventions [8]. Similarly, a reduced GSH/GSSG ratio preoperatively may indicate compromised antioxidant capacity, predicting vulnerability to oxidative stress during surgery [4]. Incorporating aminothiol profiling into preoperative assessments, alongside traditional risk factors like age and comorbidities, could refine risk models and inform surgical planning.

Monitoring oxidative stress during the perioperative period: Monitoring aminothiol levels during the perioperative period provides real-time insights into oxidative stress dynamics, enabling timely interventions. Serial measurements of plasma GSH and GSSG during CPB can detect shifts in redox balance, with a declining GSH/GSSG ratio signaling increased oxidative burden and potential organ damage [32]. Elevated homocysteine levels post-CPB may indicate ongoing inflammation and endothelial stress, guiding the use of antioxidant therapies [24]. This monitoring approach could facilitate early detection of complications like AKI or myocardial injury, allowing for prompt management to mitigate damage.

Therapeutic Implications

Potential interventions to modulate aminothiol levels: Interventions targeting aminothiol pathways offer therapeutic potential to mitigate oxidative stress in CABG patients. Antioxidant therapies, such as N-acetylcysteine (NAC), have been shown to replenish GSH levels and reduce oxidative damage during cardiac surgery, potentially decreasing the incidence of postoperative atrial fibrillation and AKI [20]. Folate or vitamin B supplementation can lower homocysteine levels, reducing its pro-oxidative and pro-inflammatory effects, which may improve long-term cardiovascular outcomes [48]. These interventions require optimization of dosing and timing to maximize efficacy, with clinical trials needed to establish standardized protocols.

Targeting aminothiol dysregulation offers promising avenues for improving outcomes in CABG patients, with interventions such as N-acetylcysteine, folate, and betaine addressing oxidative stress and homocysteine-related complications. Table 4 summarizes the potential therapeutic interventions targeting aminothiols, detailing their mechanisms of action, potential benefits in CABG, and the evidence or research needed to support their clinical adoption.

**Table 4:** Potential Therapeutic Interventions Targeting Aminothiols. ROS: reactive oxygen species; AKI: acute kidney injury; CPB: cardiopulmonary bypass; CABG: coronary artery bypass grafting; MACE: major adverse cardiovascular events; GSH: reduced glutathione; GSSG: oxidized glutathione; BHMT: betaine-homocysteine methyltransferase.

Intervention	Target Aminothiol	Mechanism of Action	Potential Benefits in CABG	Evidence/Study Needs
N-acetylcysteine	Glutathione	Replenishes GSH, enhances ROS scavenging via GSH/GSSG cycle [52].	Reduces AKI, atrial fibrillation, and oxidative stress during CPB [52].	Requires trials for optimal dosing and timing in CABG [52].
Folate/Vitamin B	Homocysteine	Reduces homocysteine via remethylation to methionine [33].	Decreases risk of atrial fibrillation, MACE, and graft restenosis [33,50].	Needs randomized controlled trials to confirm efficacy in CABG [50].
Betaine	Homocysteine	Promotes homocysteine remethylation to methionine via BHMT pathway [33].	Lowers homocysteine, potentially reducing endothelial dysfunction and MACE [33].	Limited CABG-specific data; requires clinical studies to assess impact [33].

Challenges in Translating Biomarker Research Into Clinical Practice

Translating aminothiol biomarker research into clinical practice faces several challenges, including assay standardization and cost-effectiveness. Variability in measurement techniques, such as HPLC versus MS, can lead to inconsistent results, hindering the establishment of universal reference ranges [38]. Additionally, the cost and complexity of aminothiol assays may limit their routine use in resource-constrained settings. Regulatory approval and integration into existing clinical workflows also pose barriers, requiring evidence from large-scale studies to demonstrate clinical utility and cost-benefit ratios [49].

Personalized Medicine

Role of aminothiols in tailoring treatment strategies for CABG patient: Aminothiols play a pivotal role in tailoring treatment strategies for CABG patients, enabling personalized medicine approaches. Patients with elevated homocysteine may benefit from targeted homocysteine-lowering therapies, such as folate supplementation, to reduce oxidative stress and improve graft patency [48]. Those with low GSH levels could receive NAC or other antioxidants perioperatively to bolster redox defenses and minimize organ injury [20]. Integrating aminothiol profiling with genetic and clinical data could further refine treatment plans, identifying patients who would benefit most from specific interventions. This personalized approach has the potential to optimize outcomes, reduce complications, and enhance recovery in CABG patients [53].

Challenges and limitations

The use of aminothiols as biomarkers of oxidative stress in CABG patients is promising but faces several challenges and limitations that hinder their widespread clinical application. These challenges include variability in study designs and measurement techniques, confounding factors, limited standardization of aminothiol assays, and gaps in longitudinal data and large-scale studies.

Variability in study designs and measurement techniques poses a significant challenge in interpreting aminothiol data across studies. Research on aminothiols in CABG employs diverse methodologies, including prospective cohort studies, case-control studies, and observational analyses, which differ in sample size, patient populations, and outcome measures [4]. Measurement techniques for aminothiols, such as HPLC, MS, and ELISA, vary in sensitivity and specificity, leading to inconsistent results [54]. For instance, HPLC-based methods may differ in derivatization protocols, affecting the accuracy of glutathione (GSH and GSSG) measurements, while ELISA for homocysteine may lack precision for low-abundance thiols [55]. This heterogeneity complicates meta-analyses and limits the ability to draw definitive conclusions about aminothiol levels and their clinical significance.

Confounding factors, such as patient comorbidities and medication use, further complicate the interpretation of aminothiol levels in CABG patients. Comorbidities like diabetes, hypertension, and chronic kidney disease can independently alter aminothiol metabolism, with diabetes reducing GSH levels and kidney dysfunction elevating homocysteine [56]. Medications commonly used in CABG patients, such as statins, beta-blockers, or folate supplements, can also influence aminothiol profiles; for example, folate supplementation lowers homocysteine levels, potentially masking its prognostic value [57]. These confounders make it challenging to isolate the direct effects of oxidative stress on aminothiol levels and their association with clinical outcomes.

Limited standardization of aminothiol assays in clinical practice is a major barrier to their routine use as biomarkers. Unlike established cardiovascular biomarkers like troponin, aminothiol assays lack standardized protocols for sample collection, storage, and analysis, which can lead to variability in results due to thiol oxidation or degradation [40]. For example, GSH is highly susceptible to oxidation during sample handling, necessitating immediate processing or stabilization, which is not uniformly implemented across laboratories [54]. The absence of reference ranges for aminothiols in CABG populations further limits their clinical utility, as normal versus pathological levels remain poorly defined [6].

Gaps in longitudinal data and large-scale studies represent another critical limitation. Most studies on aminothiols in CABG are cross-sectional or short-term, focusing on perioperative changes, with few investigating long-term outcomes like graft patency or MACE over extended periods [24]. The lack of large-scale, multicenter studies limits the generalizability of findings, as existing studies often involve small cohorts with heterogeneous patient characteristics [51]. Longitudinal data are needed to establish whether aminothiol dynamics predict long-term outcomes and to validate their prognostic value in diverse populations. Addressing these gaps requires prospective, well-powered studies with standardized methodologies to confirm the role of aminothiols in CABG risk stratification [8]. Table 5 summarizes these challenges, providing detailed descriptions, proposed solutions, and research needs to guide the development of robust aminothiol-based diagnostics for CABG patients.

**Table 5:** Challenges and Solutions for Aminothiol Biomarker Use in CABG. HPLC: high-performance liquid chromatography; LC-MS: liquid chromatography-mass spectrometry; ELISA: enzyme-linked immunosorbent assay; CABG: coronary artery bypass grafting; TCEP: tris(2-carboxyethyl)phosphine.

Challenge	Description	Proposed Solutions	Research Needs
Assay variability	Differences in HPLC, LC-MS, ELISA sensitivity and specificity affect measurement accuracy [37].	Standardize protocols for sample handling and analysis, e.g., use TCEP to prevent oxidation [37].	Collaborative studies to validate methods across platforms [37].
Confounding factors	Diet, medications, and comorbidities (e.g., diabetes) alter aminothiol levels [33].	Adjust for confounders in study design, use multivariate models [33].	Longitudinal studies to quantify confounder impacts in CABG [33].
Lack of standardization	No universal reference ranges or protocols for aminothiol measurement in CABG [31].	Develop consensus guidelines for reference ranges and assay protocol [31].	Multi-center trials to establish standardized cutoffs and methods [31].
Limited longitudinal data	Few studies track aminothiol changes post-CABG over time [50].	Conduct prospective cohort studies with serial measurements [50].	Long-term studies to assess prognostic value and therapeutic monitoring [50].

Future directions

The potential of aminothiols as biomarkers of oxidative stress in CABG patients is evident, but several research and clinical advancements are needed to fully realize their utility. Future directions include developing standardized protocols for aminothiol measurement, integrating aminothiol profiling into personalized medicine, exploring therapeutic interventions targeting aminothiol pathways, and leveraging emerging technologies such as metabolomics to advance biomarker research.

Standardized protocols for aminothiol measurement are essential to enhance the reliability and reproducibility of results in clinical practice. Current measurement techniques, such as HPLC and MS, suffer from variability due to differences in sample handling, derivatization methods, and assay conditions, particularly for labile compounds like GSH [38]. Establishing uniform guidelines for sample collection, storage, and analysis, along with defining reference ranges for aminothiols in CABG populations, would facilitate their integration into routine clinical diagnostics [6]. Collaborative efforts among research institutions and clinical laboratories are needed to develop and validate these protocols, ensuring consistency across studies and settings.

Integrating aminothiol profiling into personalized medicine holds significant promise for improving CABG outcomes. Preoperative aminothiol levels, such as elevated homocysteine or reduced GSH, could identify high-risk patients who may benefit from tailored interventions [8]. For example, patients with hyperhomocysteinemia might receive folate or vitamin B12 supplementation to lower homocysteine levels, potentially reducing postoperative complications like atrial fibrillation [57]. Similarly, GSH profiling could guide antioxidant therapy to mitigate oxidative stress during surgery. Incorporating aminothiol data into risk stratification models, alongside other biomarkers like troponin, could enhance precision in predicting adverse outcomes and optimizing patient management [4].

Therapeutic interventions targeting aminothiol pathways represent a key area for exploration. Antioxidant supplementation, such as NAC, has shown potential in replenishing GSH levels and reducing oxidative stress in cardiac surgery patients [20]. Clinical trials are needed to evaluate the efficacy of NAC or other thiol-modulating agents in improving short-term outcomes, such as reducing acute kidney injury, and long-term outcomes, like graft patency [24]. Additionally, targeting homocysteine metabolism through folate or betaine supplementation could mitigate its pro-oxidative effects, potentially lowering the incidence of MACE [57]. These interventions require rigorous testing to establish optimal dosing, timing, and patient selection criteria.

Emerging technologies, particularly metabolomics, offer exciting opportunities to advance aminothiol biomarker research. Metabolomics enables comprehensive profiling of aminothiols and related metabolites, providing insights into the broader redox network and its dynamics during CABG [53]. High-resolution MS-based metabolomics can detect subtle changes in low-abundance thiols like cysteinylglycine, improving the sensitivity and specificity of biomarker panels [58]. Integrating metabolomics with machine learning could identify novel aminothiol-related signatures associated with specific CABG outcomes, facilitating the development of predictive models [59]. These advancements could transform aminothiol research, moving it toward clinical applications that enhance patient care.

## Conclusions

The exploration of aminothiols as biomarkers of oxidative stress in coronary artery bypass grafting (CABG) underscores their significant potential in enhancing perioperative care and improving patient outcomes. Glutathione, homocysteine, cysteine, and cysteinylglycine play critical roles in maintaining redox homeostasis, with their dysregulation reflecting the oxidative burden induced by ischemia-reperfusion injury and cardiopulmonary bypass during CABG. These molecules offer valuable insights into the pathophysiology of postoperative complications, such as atrial fibrillation, acute kidney injury, and myocardial injury, as well as long-term outcomes, including graft restenosis and major adverse cardiovascular events.

The evidence suggests that preoperative and perioperative aminothiol profiling can facilitate risk stratification, enabling clinicians to identify high-risk patients and tailor interventions, such as antioxidant therapies or homocysteine-lowering strategies, to mitigate oxidative stress. However, challenges such as assay variability, lack of standardization, and confounding factors like comorbidities and medications limit their current clinical utility. Future research should focus on developing standardized measurement protocols, conducting large-scale longitudinal studies, and leveraging advanced technologies like metabolomics to refine aminothiol-based diagnostics. By addressing these challenges, aminothiols could become integral to personalized medicine in CABG, paving the way for targeted therapeutic strategies that enhance recovery and reduce morbidity and mortality in this patient population.
